# Empirically testing a relationship between cooperation and the prime numbers

**DOI:** 10.1098/rsos.231425

**Published:** 2024-06-19

**Authors:** Tim Johnson

**Affiliations:** ^1^ Atkinson School of Management, Willamette University, Salem, OR, 97301, USA

**Keywords:** cooperation, prime numbers, public goods game, placebo tests

## Abstract

Theoretical models suggest a relationship between cooperation and the prime numbers. In environments where agents play multiple one-shot prisoner’s dilemma games per generation, cooperators evolve to fixation more frequently when cooperating on a cyclical schedule with a prime-number period length. This finding parrots classic predator–prey models showing selection for prime-number prey life cycles. Here, I report an empirical test of the former models using previously published data concerning humans playing one-shot public goods games across multiple time points—i.e. an analogue to multiple one-shot prisoner’s dilemma games. I find very modest evidence of cyclicality at prime-numbered time intervals, though results indicate rough agreement between theoretical predictions and observed rates of full cooperation across time points. Analyses of individual decisions find increased contributions to the public good at prime-number time points and separate placebo tests indicate a 4-in-1000 chance of spuriously estimating this effect. However, when exploratory analyses exclude low-value prime-numbered time points, the magnitude of the estimated effect decreases and the hypothesis of no effect cannot be rejected, implying that low-value, prime-number time points drive estimates, contrary to theoretical model predictions. These findings cast doubt on the hypothesis of increased cooperation at prime-number time points—at least among humans playing public goods games.

## 1. Introduction

Cooperation—i.e. the individual decision to incur costs that benefit others even when those others might avoid such costs through free-riding—is central to life [1–4]. Not only does cooperation manifest among diverse life forms engaged in various activities (from bacterial production of siderophores [5] to human use of irrigation [6]), but it also plays a role in individuals’ aggregation into larger-scale entities, such as multi-cellular organisms or social groups [2–4,7–9]. Due to these substantive implications and its venerated position in human social experience [3,8,9], cooperation remains a focus of interdisciplinary inquiry and this intellectual diversity appears vividly in the wide range of fields that have uncovered ways that cooperation can evolve. Cooperation can result from genetic relations studied in biology [10–12], network properties learned from physics [13–18] and institutional structures conceptualized in the social sciences [8,19–23]—not to mention from means discovered at the intersection of scientific disciplines, such as mobility [24–26], reciprocity [1,27–30] and cues indicating cooperative dispositions [31–34].

Recent theoretical models [35,36] add both to this list of ways that cooperation can evolve and to the academic variety of this research area. The models show that cyclical behaviour on prime-number period lengths (as observed in predator–prey models predicting prime-valued prey life cycles [37,38]) can instigate cooperation’s evolution by allowing cooperators to evade defectors who spread themselves across a large number of composite time points [35,36]. Such findings establish how a formative mathematical construct (the prime numbers) connects to models of cooperation as foreshadowed in past research [37], thus branching off from a more general line of inquiry that studies the mathematical underpinnings of moral behaviour (i.e. not solely cooperation) [39,40]. In so doing, the results add further evidence to a genre of research showing connections between models of natural processes and prime numbers [37,41–43].

Indeed, findings from a realistic, computationally implemented theoretical model [36] give reason to examine the possibility that a relationship between prime numbers and cooperative behaviour might manifest in empirical data. The model in [36] studied the evolution of cyclical strategies when simulated agents played multiple, one-shot prisoner’s dilemma games per generation and strategies evolved according to well-known selection processes. This article reports an empirical test of that possibility. The test studies previously published data from a public goods game experiment conducted by Gächter *et al*. [44] that contained a condition reminiscent of the aforementioned model’s depiction of agents playing in multiple, one-shot prospectively cooperative social encounters. In the condition, participants played one-shot public goods games over 27 time points (or ‘rounds’) without behavioural options such as punishment [19,45,46] or reputational information [47]. A well-known decline in contributions occurs with successive decisions in experiments where individuals play multiple one-shot games without a behavioural option that supports cooperation; this decline is due to the presence of defectors and reciprocators who conditionally cooperate [44]. However, sporadic cooperation appears at low rates even in late rounds, thus I explore whether the aforementioned prime-numbered cycles might explain this ongoing cooperation. The data from this condition of Gächter *et al*. [44] offered a unique opportunity to study the relationship between prime numbers and cooperation because it (i) involved decision-making in a scenario structurally analogous to the prisoner’s dilemma and (ii) arranged this decision-making across a larger-than-normal number of time points in one-shot versus repeated encounters. In the subsequent two paragraphs, I elaborate on features (i) and (ii) of the experimental condition, from Gächter *et al*. [44], which I investigate.

With respect to feature (i), researchers have long recognized the structural similarities between the public goods game and the prisoner’s dilemma [48], and they have regarded the former as a multi-person variant of the latter [49]. Both games create a face-off between group-benefiting action (i.e. contribution in the public goods game and cooperation in the prisoner’s dilemma) and the self-interested choice of free-riding (i.e. withholding contribution in the public goods game and defecting in the prisoner’s dilemma). However, the scenarios do exhibit differences: the prisoner’s dilemma presents a stark binary choice to cooperate or defect, whereas the public goods game lets players vary the degree of cooperation by contributing amounts that range from nothing to full cooperation, with partial amounts in between. In the empirical investigation that I report here, I study both full cooperation and granular contributions reported in Gächter *et al*. [44]. This approach attempts to balance both the similarities and differences that exist between the prisoner’s dilemma model (which figures centrally in the theoretical foundation for the present empirical analysis, viz. [36]) and the public goods game (which is the experimental paradigm used in Gächter *et al*. [44]).

With respect to feature (ii), Gächter *et al*. [44] report unique data in the sense that experiments rarely examine decision-making in potentially cooperative social encounters that both occur over a substantial number of time periods and are ‘one-shot’ (versus games that occur between the same individuals over multiple rounds—i.e. repeated games [28,50–53]). Gächter *et al*. [44] study behaviour over a sequence of 27 decisions, which I refer to variously as time points and rounds, thus affording the present investigation with the opportunity to examine behaviour across a substantial number of prime-valued increments (e.g. the second, third, fifth time points and so on). Also, the experimental condition of Gächter *et al*. [44] shuffles participants across groups in each round of the experiment, thus meaning that participants do not have reason to believe that they participate with the same individuals (i.e. in a repeated game) across time points of the experiment. This implementation of a one-shot public goods game also approximates the one-shot nature (i.e. random assignment of partners) in the theoretical models whose predictions I propose to test in the present empirical study (i.e. [36]).

Given these features, the data from Gächter *et al*. [44] provide a unique opportunity to test the hypothesis of cyclical cooperation with prime-valued period lengths. I study the data in both the frequency and time domain. That is, in the frequency domain, I report analyses that examine whether cyclicality exists in the present investigation’s empirical data and whether any such cycles exhibit prime-numbered period lengths as hypothesized in the theoretical models that I test. In the time domain, I perform a fresh run from the basic computational model in [36] and then compare patterns in the simulated data with patterns in the aggregate experimental data; I also repeat the regression analysis of Gächter *et al*. [44] with the addition of model terms capturing the potential effect of prime-number time points (or ‘rounds’). I further augment these analyses with placebo tests and exploratory analyses that challenge the robustness of my investigation’s findings.

Results reported here show little evidence of cyclicality at prime-numbered time intervals, yet aggregate data from the experimental condition in Gächter *et al*. [44] roughly agree with the output of computational models showing selection for cooperation on prime-number cycles. Also, regression analyses suggest increased cooperation at prime-number time points. Subsequent exploratory analyses, however, indicate that low-value prime periods drive this estimated effect. This latter finding departs from theoretical predictions, thus raising questions about the hypothesis of cooperation at prime periods—at least among humans playing the public goods game.

## 2. Results

Consistent with evidence reported in [44], contributions declined across the experiment’s 27 periods: average contributions of 6.53 tokens (s.d. = 7.14) in period 1 decreased to average contributions of, respectively, 1.64 tokens by period 9 (s.d. = 3.63) and 1.07 (s.d. = 3.18) tokens by period 27. The median contribution declined to 0 by period 3 and remained at nil for all subsequent periods.

**Figure 1.**
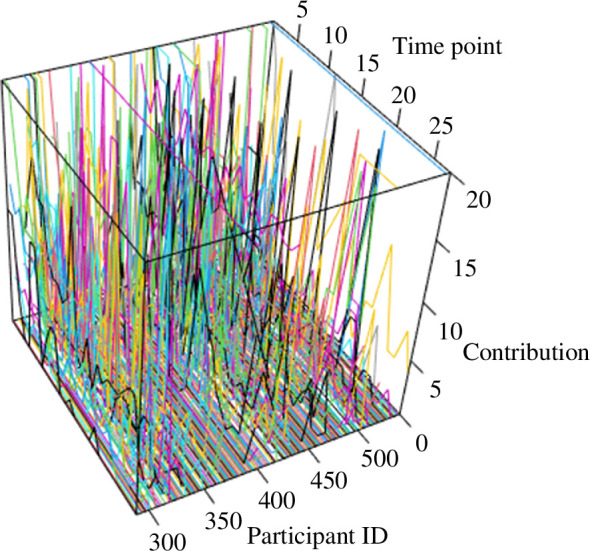
Participants’ contributions generally decline over time, but a portion of the participant pool continues to contribute even in later rounds. Figure 1 displays data from the condition in Gächter *et al*. [44] in which participants played multiple one-shot public goods games across 27-time points without any sanctioning institution or reputational mechanisms. Each participant’s series of contribution decisions (vertical axis) across time points (horizontal axis 1) appears as a line in the three-dimensional plotting field [54], with participants’ anonymous identification number demarcating each series (horizontal axis 2). The erratic nature of the series displayed in the figure speaks to the variation in contribution behaviour that occurs across time points (even those later ones) and the potential for cyclicality that exists among a fraction of the participant pool, thereby making a test for cycles with prime-valued periods reasonable.

This main trend of declining contributions masks variation in contributions that persists in late rounds of the experiment, however. Figure 1 attests to this variation by showing a cacophony of jagged data series that stand in contrast to the common trend of declining contributions. Likewise, though the percentage of participants contributing more than 0 declines from approximately 59% (150 participants) in round 1 to 30% (77 participants) in time point 9, almost one-quarter of the participants (about 24%; 61 participants) contribute 1 or more tokens in round 18 and 21% (53 participants) do so in the last time point, round 27. Thus, among some participants, contributions continue even as the overall contribution trend declines across time points.

The persistence of cooperation into late rounds raises the possibility that some participants might deploy a strategy akin to cyclical strategies with prime-number period lengths that facilitate the evolution of cooperation in the theoretical models reported in [36]. That is, although the presence of consistent defectors and reciprocators who conditionally cooperate leads to declining contributions, some fraction of participants continue to cooperate in defiance of that overall trend of declining contributions and, among this fraction, cyclical strategies of the variety studied in [36] remain a not implausible model of contribution behaviour. As a result, I analysed autocorrelation in each participant’s series of decisions and identified the lag with the maximum autocorrelation (if it existed) for each participant in the data. Out of the 256 participants’ time series, 32 series (12.5%) exhibited a maximum autocorrelation with a prime-valued lag. These series with prime-valued lags amount to 24.6% of the 130 series of contributions that were neither monotonic across time points nor always 0. These results provide very modest evidence of cyclical contributions with prime-number periods.

**Figure 2.**
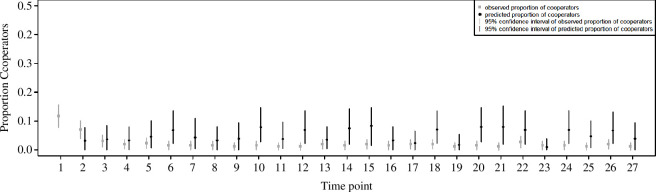
Comparison of observed data from public goods experiments (grey elements) with simulation output from the theoretical model (black elements). After producing 10^4^ fresh runs of the evolutionary simulation from [36], I took a random draw of 256 runs (i.e. a draw equalling the number of subjects (*n* = 256) in the relevant condition of [44]) and identified the behaviour-schedule strategy pair that grew to fixation in each run. Please note that only one behaviour-schedule strategy grows to fixation in a particular simulation. I then simulated the behaviour of the strategy across 27 periods, as if the strategy were a participant in [44] (see §4) to create the theoretically predicted proportion of cooperators. This value varied with each random draw of strategies that grew to fixation because the composition of strategies changed stochastically with each draw. I next stored the predicted proportion of cooperators in each period of the simulated experiment. I repeated these procedures 10^4^ times to create a sampling distribution of such predictions in each time point (save for time point 1 in which the theoretical model makes no predictions). The mean of this sampling distribution, in each period, appears as the black dot in figure 2; its 95% CI appears as the black line. The grey squares denote the empirically observed proportions of cooperators in each period of the relevant experimental condition from [44] (the ‘strangers’ condition) and the grey lines denote their 95% CIs. The figure provides evidence suggesting a very rough agreement between simulation results and the empirical data. That is, the simulation results reside in the same region of the plotting field and CIs regularly overlap, but point estimates do not exhibit a comparable pattern.

In addition to studying periodic behaviour in the frequency domain, the current analysis also explored the time domain. Simulations of the model presented in [36] largely captured aggregate patterns of cooperation in the empirical data across time points. In fresh simulation runs with a maximum possible time point set to 27 in order to match the experimental data (see §4), I found that the 95% confidence intervals (CIs) of the predicted proportions of cooperators in each round overlapped with the 95% CIs of the observed proportion of full contribution decisions in the empirical data (see figure 2).

In addition to this rough agreement between theoretical model output and empirical data, analyses of individual decisions indicated increased cooperation at prime-number time points. In the midst of declining contributions over the course of the experiment, participants contributed an average of 2.28 tokens (s.d. = 4.55) to the public good in prime-number rounds and an average of 1.71 tokens (s.d. = 4.06) in composite-number rounds, thus rejecting the null hypothesis of no difference in contributions between prime- and composite-valued time points (Wilcoxon rank sum test; *W* = 5.65 × 10^6^, *p* = 7.74 × 10^−8^, two-tailed).

These observed differences, however, might reflect concurrent declines in (i) participants’ contributions across time points and (ii) the prime numbers’ frequency over the stretch of the number line under study (e.g. four primes appear in the first nine positive integers, three primes appear in the next nine, and two appear in the final nine). As a result, I estimated regression models that accounted for declining participant contributions across time. I created a binary indicator signalling whether a decision took place in a prime-number time point and added this indicator to the regression model reported by Gächter *et al*. [44]. The regression model from [44] depicted participants’ contributions in a given round as a function of the experimental round’s numerical value, the lagged average contributions of a participant’s group mates in the previous round, and random intercepts for participants and matched groups. Even when statistically accounting for these covariates and sources of clustering, the estimated coefficient for the prime-time-point indicator took a positive value and was estimated with sufficient precision to reject the null hypothesis that it equalled 0 ( $\hat{\beta }=0.218$ , s.e. = 0.08, *z* = 2.82, *p* = 0.005, 95% CI = [0.07, 0.37], *n* = 6656 participant-period decisions; see electronic supplementary material for all estimated coefficients and relevant statistics). Furthermore, the substantive and statistical interpretation of the estimated coefficient for the prime-time-point indicator remained unchanged across model specifications that serially removed covariates (see electronic supplementary material).

To explore the possibility that the value and CI of estimated coefficients resulted spuriously from properties of the prime-time-point indicator, I performed placebo tests using a novel method that replaced prime numbers with composite numbers distributed akin to the primes. That is, the estimated coefficient of the prime-time-point indicator might exhibit a magnitude and precision that does not reflect an effect related to the exact location of the primes on the number line *per se*, but rather more general features of the primes’ distribution along the number line. For instance, such a spurious relationship might result from the total number of primes in a given span of the number line. To address this problem, one can define sets of other numbers that exhibit such properties of the primes even if they are not prime and study whether they yield comparable effect estimates when substituting for the primes in the present empirical analysis. For instance, four primes appear in the first 10 integers, thus one could define the set *A =* {4,6,8,9} so as to manufacture a set that shares an attribute of the prime’s distribution, even though no element of the set is a prime number. The many such sets of this type then could serve as placebos in repetitions of the above-presented statistical analyses to determine the rate of obtaining estimates of a magnitude and precision comparable to those reported above. Using these sets of ‘placebo primes’, I created a ‘placebo-prime-time-point indicator’ that took a value of 1 when a contribution decision was made in one of the placebo-prime time points (taking the value 0 otherwise) and replaced the prime-time-point indicator with this placebo-prime-time-point indicator in the focal regression model, which was then re-estimated. I repeated this procedure for all possible sets of placebo primes, producing thousands of estimates of the placebo-prime-time-point indicator whose values and CIs could be compared with the estimates of the prime-time-point indicator in the focal regression model.

The first set of placebo tests focused on the total count of primes that appear across the first 27 positive integers (i.e. nine primes), thus it constructed all 24 310 possible sets of nine composite numbers from the interval spanning 1 to 27 (inclusive) and treated each as a set of placebo primes. It then used each set of placebo primes to create the placebo-prime-time-point indicator and repeated the focal regression analysis with the placebo-prime-time-point indicator replacing the prime-time-point indicator. These placebo tests produced a positive coefficient, with the lower bound of its 95% CI greater than 0, in 88 of the 24 310 (0.36%) iterations.

An additional two placebo tests sought to account for the uneven distribution of the prime numbers across the interval spanning from 1 to 27. These tests tallied the number of primes across ‘bins’ that divided the interval into, respectively, two and three parts; then, the tests created all possible sets of placebo primes that matched the frequency distribution of primes in those bins.

Placebo tests using two bins split the interval spanning 1 to 27 into an interval stretching from 1 to 14 and another from 15 to 27 (all intervals inclusive). It then created sets of placebo primes that reflected the number of primes in each of those two bins—namely, six in the first bin and three in the second bin—and, then, it repeated the current investigation’s regression analysis for each of these 840 sets of placebo primes. Not once, in the 840 placebo tests involving two bins, did the placebo-prime-time-point indicator’s coefficient take a positive value, let alone possess a lower bound of its 95% CI resting to the right of 0.

When splitting the interval 1 to 27 into three bins, four primes appear among the first nine integers on the interval, three primes among the next nine integers, and two primes among the final nine integers; thus, the placebo test with three bins formed sets of composite numbers that included four composites from the integer values spanning 1 to 9, three composites from the values spanning 10 to 18, and two composites from the values stretching from 19 to 27 (all inclusive). I then recreated the placebo-prime-time-point indicator for each of the 420 sets of these ‘three-binned’ placebo primes and repeated the focal regression analysis, iteratively, using each of those placebo-prime-time-period indicators in place of the prime-period indicator. As in the test using two bins, 0 out of the 420 placebo tests involving three bins yielded an estimated coefficient of the placebo-prime-time-point indicator that was positive, thus the lower bound of the estimates’ 95% CIs never exceeded 0.

**Figure 3.**
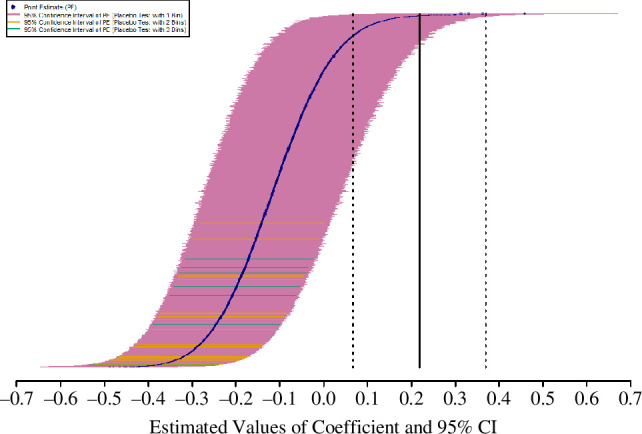
Point estimates and 95% CIs of placebo tests show a low probability of obtaining a positive estimate with a 95% CI resting to the right of 0. The figure displays the point estimate (navy blue dot) and 95% CI of each placebo test (pink, orange and turquoise lines). Placebo tests involve the creation of a set of nine composite numbers that my investigation labels the ‘placebo primes’ (i.e. a set of composites with the same number of elements as the number of primes in the interval [1,27]); in a test with one bin, the set can include placebo primes from any span of the interval [1,27], whereas placebo tests with two bins form sets of placebo primes with the restriction that the number of composites drawn from the intervals [1,14] and [15,27] match the number of primes in those spans and those with three bins form sets of placebo primes in which the number of composites drawn from the intervals [1,9], [10,18] and [19,27] equals the number of primes in those intervals. The observed point estimate for the prime-period indicator’s coefficient in the present investigation’s focal regression model appears as the solid black vertical line; it is flanked by dashed vertical lines on the left and right that represent, respectively, the 95% CIs lower- and upper-bounds for the prime-period indicator’s estimated coefficient. The figure shows that in the vast majority of placebo tests, the placebo-prime indicator very rarely exhibited a positive coefficient with a lower-bound CI greater than 0. These results conflict with the concern that the positive, statistically significant estimates of the prime-round indicator were spurious.

**Figure 4.**
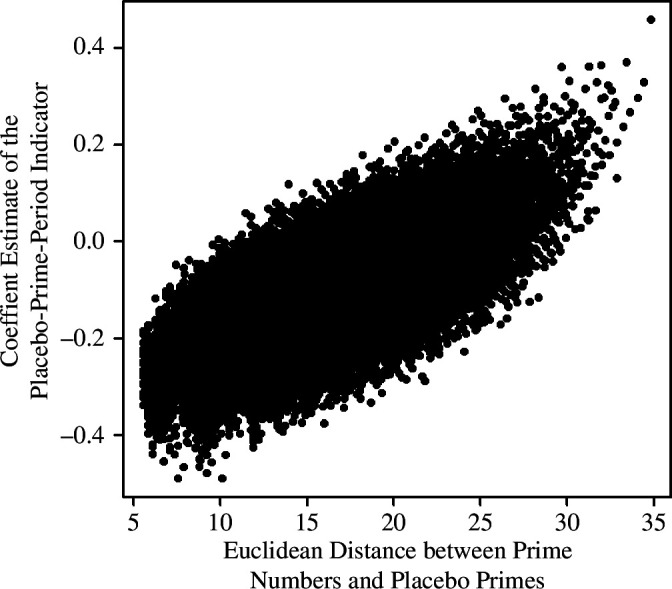
Positive relationship between the prime numbers’ Euclidean distance from placebo primes and the estimated coefficient values of the placebo-prime-time-point indicator (referred to as ‘the placebo-prime-period indicator’ for concision on the vertical axis) in placebo tests. Multiple bins force the placebo primes to congregate more closely to the actual primes, thus decreasing Euclidean distance and potentially accentuating a contrast between the greater contributions to the public good in prime-number time points versus the lower contributions in non-prime rounds. Plotting coefficient estimates from placebo tests as a function of the Euclidian distance of each set of placebo primes from the prime numbers corroborated this phenomenon.

In total, across all 25 570 placebo tests, only 88 yielded a coefficient for the placebo-prime-time-point indicator that exceeded the value of the focal regression analysis’ prime-time-period indicator and only 300 tests (1.17%) possessed a positive coefficient with a lower-bound of the 95% CI greater than 0 (see figure 3). Although these findings offer some support for the notion that the results of the focal regression analysis were not spurious, they also sparked curiosity: the lack of positive coefficients when the placebo primes must conform to two or three bins, versus when they are drawn from one bin (i.e. the full interval), implies a relationship between coefficient estimates and the Euclidean distance between placebo primes and the actual primes. After all, multiple bins force the placebo primes to congregate more closely to the actual primes, thus decreasing Euclidean distance and potentially accentuating a contrast between the greater contributions to the public good in prime-number time points versus the lower contributions in non-prime time points. Plotting coefficient estimates from placebo tests as a function of the Euclidian distance of each set of placebo primes from the prime numbers (figure 4) corroborated this suspicion.

Examining sets of placebo primes with both large Euclidean distances from the prime numbers and large coefficient estimates indicated that those placebo primes took relatively high values, thus inspiring exploratory analyses that considered the possibility that the prime-period indicator’s coefficient estimate in the focal regression might be driven by low prime-numbered periods. Indeed, when excluding decisions from the second round from the regression analysis, the estimated coefficient of the placebo prime indicator decreases to 0.13 (s.e. = 0.07, *z* = 1.72, *p* = 0.085, 95% CI = [−0.02, 0.27], *n* = 6400), thus preventing rejection of the null hypothesis of no effect with 95% confidence. When excluding decisions from both the second and third time points, the regression analysis yielded a coefficient estimate that rests close to 0 and its 95% CI straddles 0 ( $\hat{\beta }=0.02$ , s.e. = 0.07, *z* = 0.30, *p =* 0.768, 95% CI = [−0.12, 0.16], *n =* 6144), which prevents rejection of the null hypothesis of no effect. This finding implies that low-value prime time points underlie the focal regression results.

## 3. Discussion and conclusion

The present empirical investigation produced mixed results for the hypothesis of increased cooperation at prime-number periods, thus raising questions about the hypothesis’ veracity—at least as it applies to humans playing the public goods game. Initial evidence seemed to be consistent with the hypothesis. Very modest evidence of cycles with prime period lengths appeared and aggregate empirical data roughly agreed with results from the theoretical model’s simulation output. Furthermore, analyses indicated increased contributions to the public good at prime-valued time points even when statistically controlling for confounders, not to mention that placebo tests indicated a low chance (approximately 4 in 1000) of the results being spurious. However, exploratory analyses that excluded low-value prime periods indicated that the initial results were driven by those periods, which conflicts with the results of theoretical models that show cooperators growing to fixation by implementing their pro-social behaviour at prime-number time points of both small and large values. Moreover, these results support a clear alternative hypothesis—namely, that rounds 2 and 3 happen to be both prime-numbered rounds and rounds in which participants intending cooperation still harbour some hope that others might contribute.

Accordingly, the findings in this article raise doubts about a link between the prime numbers and cooperation in human public goods games. Replication of the current findings will provide an opportunity to test that possibility further. Future empirical research on the subject also might consider examining human cooperative behaviour in other contexts—for instance, dyadic prisoner’s dilemmas—or in other organisms for which a non-intentional, cyclical strategy involving limited cognition—such as cooperating at the primes—is more plausible. The mixed evidence in this paper casts doubt on a link between cooperation and the primes in human public goods games and offers a reason for tests of the hypothesis in other settings.

## 4. Methods

### 4.1. Use of replication data from a published public goods game experiment

The present investigation uses data from Gächter *et al*. [44], a laboratory experiment that used real monetary incentives to measure the effect of framing a public goods game as either an act of (i) providing the public good (‘provision’ treatment) or (ii) refraining from extracting from a public good (‘maintenance’ treatment). In each treatment, participants reside in groups of four participants per round and make decisions over tokens that convert to UK £ after the experiment. In the provision treatment, participants receive an endowment of 20 tokens and can contribute any share of these tokens to a ‘group project’ (i.e. choose a contribution value $c_{i}$ , such that $0{\leq c}_{i}\leq 20$ ); the total sum of tokens to the group project is multiplied by 0.4 and shared equally among participants, thus yielding a pay-off function for participant i of


(4.1) $$\pi_{i}=20-c_{i}+0.4\sum_{j=1}^{4}c_{j},$$

where j indexes group members. In the maintenance treatment, participants receive no endowment of tokens but can take up to 20 tokens from a ‘group project’ that already contains 80 tokens (i.e. the participant chooses a withdraw, $w_{i}$ , such that $0{\leq w}_{i}\leq 20$ ); afterwards, the total sum of tokens left in the group project is multiplied by 0.4 and shared equally among participants, thus yielding a pay-off function for participant i of


(4.2) $$\pi_{i}=w_{i}+0.4\sum_{j=1}^{4}c_{j},$$

where j again indexes group members. The monetary incentives across these treatments remain equivalent, as inspection of the pay-off functions in equations (4.1) and (4.2) indicates [44].

Gächter *et al*. [44] investigated these treatments across four conditions: a one-shot public goods game, a ‘strangers’ condition in which participants played the public goods game in randomly reshuffled groups in each of 27 rounds, a ‘partners’ condition in which participants played the public goods game with the same group mates in each of 27 rounds, and a ‘partners with punishment’ condition in which participants played the public goods game with the same group mates in each of 27 rounds with the option to punish group mates at a cost after each period’s contribution decision was made. Given that the theoretical models tested in the present study involved randomly formed, potentially cooperative interactions across time without additional behavioural options, the ‘strangers’ condition (*n* = 256) is the relevant condition for testing hypotheses derived from the theoretical model. Accordingly, the present study uses data from the ‘strangers’ condition of the laboratory public goods experiments reported in Gächter *et al*. [44].

### 4.2. Comparison of simulation results with aggregate data

To assess the overall plausibility of deriving empirical predictions from past computational models, I examined whether the simulations’ aggregate results reasonably reflected patterns of aggregate behaviour in the empirical data. To do so, I executed new runs of the simulation from [36] but extended the maximum intergenerational time point in the simulation to 27 to reflect the number of time points over which participants played the public goods game in the experiment that serves as this study’s empirical data [44].

In the computer simulation, a finite population of agents play prisoner’s dilemma games that occur across a set of intra-generational time points; agents implement behavioural strategies of cooperation or defection in those games according to a cyclical schedule with a set period length. Evolutionary selection, in the form of a pairwise comparison process, operates on agents’ behavioural-schedule pairings over a maximum of 10^5^ generations. A run of the simulation terminates when one such pair grows to fixation and the simulation repeats for 10^4^ runs (please see the electronic supplementary material for a detailed description of the computer simulation and for access to the computer code used to execute the simulation).

The present study uses the behavioural-schedule pairings that grow to fixation across runs of the simulation as a population from which to sample ‘subjects’ whose behaviour in a simulated version of the empirically studied experiment can be compared with the behaviour of actual participants. Specifically, the current investigation randomly samples 256 runs of the evolutionary simulation (i.e. a sample size equal to the number of participants, *n* = 256, in the strangers’ condition of [44]) and regards the behavioural-schedule strategy that evolved to fixation in each run as a subject. It then simulates the decision-making of the subject across time points 2–27 by executing the behavioural strategy of the subject (i.e. full contribution for cooperators and nil contributions for defectors) at the time points dictated by the subject’s schedule. This overall procedure—that is, sampling 256 runs of the evolutionary simulation and then simulating the behaviour of strategies that grew to fixation in each of those 256 runs—repeats 10^4^ times to produce a sampling distribution of the predicted proportion of fully cooperative plays (a.k.a. the predicted proportion of cooperators) at each time period of the public goods game experiment. One can think about that procedure as treating each strategy that grows to fixation in a simulation as a potential experimental participant. From that population of experimental participants, I then draw a sample of 256 such ‘participants’—that is, a total equalling the number of participants from the condition of [44] that is studied in this article. I then simulate the behaviour of those strategies across each time point. This procedure repeats 10^4^ times and since the 256 strategies drawn in each sample can differ, the rate of cooperation across time points in each run can also differ, thus creating the sampling distribution whose rate of cooperation is then compared with the empirical data. That is, I then compared, in each round, the sampling distribution’s 95% CI of the predicted proportions of cooperators with the empirical data’s 95% CI of the observed proportion of full contribution decisions.

### 4.3. Focal regression analysis

To account for co-varying factors that might confound the effect of prime-number periods on individual contribution decisions, I add the prime-time-point indicator to the model of individual contribution decisions reported in [44] (viz. figure 3, and S.M., p. 4), which aimed to depict the effect of reciprocity and declining cooperation across time on contributions. Specifically, quoting from [44] (with the addition of the prime-time-point indicator), the model takes the form


(4.3) $$c_{i,j,t}=\beta_{0}+u_{0m}+u_{0i}+\beta_{1}{\bar{c}}_{-i,j,t-1}+\beta_{4}Round+\beta_{5}Prime+\varepsilon_{i,j,t},$$

such that ‘ $c_{i,j,t}$ is the effective contribution of individual i in group j at round t ; $\beta_{0}$ is a constant; $u_{0m}$ and $u_{0i}$ are random intercepts at the matching group and individual level, respectively. ${\bar{c}}_{-i,j,t-1}$ is the average contribution of the other three group members from the previous round. The variable Round indicates the round of the experiment and estimates a time trend’ (again, quoting from [44], S.M., p.4). The variable Prime is the prime-time-point indicator, which is a binary indicator that takes a value of 1 if t takes a prime-number value and a value of 0 otherwise. The present study estimates this model, first, on the full dataset from the ‘strangers’ condition in [44], and then it estimates the model repeatedly in placebo tests described in §4.4.

### 4.4. Placebo tests

To gauge the possibility that the value and statistical significance of estimated coefficients resulted spuriously from properties of the prime-time-point indicator, I performed placebo tests that replaced prime numbers with composite numbers that it labelled ‘placebo primes.’ Construction of the placebo primes sought to capture features of the prime numbers’ distribution across the first 27 positive integers and it took three forms.

The first placebo test noted that nine prime numbers appear across the first 27 positive integers, thus it constructed all possible sets of nine composite numbers from the interval spanning 1 to 27 and regarded each set of composites as a set of placebo primes. Then, for each of these 24 310 sets of placebo primes, the placebo test created a new prime-period indicator (hereafter, the ‘placebo-prime-time-point indicator’), which took a value of 1 when a contribution decision occurred in a round equal to the value of a placebo prime. The placebo test subsequently repeated the focal regression analysis 24 310 times, with each iteration substituting a placebo-prime-time-point indicator for the prime-time-point indicator and re-estimating the focal regression model.

An additional two placebo tests sought to account for the uneven distribution of the prime numbers across the interval spanning from 1 to 27. These tests tallied the number of primes across ‘bins’ that divided the interval into sets of, respectively, two and three, then the tests created all possible sets of placebo primes that matched the frequency distribution of primes across the bins. For instance, when dividing the interval spanning 1–27 into three bins, the present investigation tallied four primes among the first nine integers on the interval, three primes among the next nine integers and two primes among the final nine integers in the third bin; the test then formed all sets of composite numbers that included four composites from the integer values spanning 1 to 9, three composites from the values spanning 10 and 18, and two composites from the values stretching from 19 to 27. The placebo test then created a placebo-prime-time-point indicator for each of these sets of ‘binned’ placebo primes and serially re-estimated the focal regression model with each ‘binned’ placebo-prime-time-point indicator substituted for the prime-time-point indicator (i.e. a new regression was performed for each placebo-prime-time-point indicator). The same procedures were performed for a test that split the interval spanning 1–27 into two bins (one stretching from 1 to 14 and the other from 15 to 27, each inclusive) and created sets of placebo primes that reflected the number of primes in each of those two bins (i.e. six values in the first bin and three values in the second bin, for a total of nine numbers in the set).

### 4.5. Exploratory analysis of placebo results and dropping primes

To understand the variation in coefficient estimates generated in the placebo tests, I computed the Euclidian distance between the prime numbers and a given set of placebo primes, then plotted this distance against the estimated coefficient from the regression estimated using those placebo primes. This procedure was repeated for each set of placebo primes. The resulting plot indicated a positive correlation between distances and coefficient values, thus raising the suspicion that particular prime-number rounds drive the results; that is, the primes congregate at relatively low values in the stretch of the number line from 1 to 27, and the positive correlation suggests that low-valued composite periods (i.e. closer to more primes) buttress the estimated coefficient value of the prime-period indicator whereas higher-valued composite periods (i.e. further from the primes) suppress the coefficient value because they exhibit comparable levels of contributions as higher-valued prime periods. To explore this intuition, I created a subset of the data consisting of all time points greater than round 2 and another subset consisting of all time points greater than round 3; it then replicated the focal regression analysis on each of these subsets of data.

## Data Availability

Complete computer code to perform this article’s analyses in R [55], as well as select analyses in STATA for comparison with [44], can be found at the OSF project page for this article: https://osf.io/2fkya/. Experimental data used in the present investigation were made publicly available by the authors of [44], prior to the commencement of the present study, can be downloaded for replication purposes [56]. Other datasets used in the completion of this project can be found on the aforementioned OSF project page. Supplementary material is available online [57].
